# Peptidylarginine deiminase (PAD) is a mouse cortical granule protein that plays a role in preimplantation embryonic development

**DOI:** 10.1186/1477-7827-3-42

**Published:** 2005-09-01

**Authors:** Min Liu, Andrea Oh, Patricia Calarco, Michiyuki Yamada, Scott A Coonrod, Prue Talbot

**Affiliations:** 1 Department of Cell Biology and Neuroscience, University of California, Riverside, California 92521, USA; 2 Department of Anatomy and Medicine, School of Medicine, University of California, San Francisco, California 94143, USA; 3 Graduate School of Integrated Science, Yokohama City University, Yokohama, 236-0027 Japan; 4 Weill Medical College of Cornell University, New York, NY 10021, USA

## Abstract

**Background:**

While mammalian cortical granules are important in fertilization, their biochemical composition and functions are not fully understood. We previously showed that the ABL2 antibody, made against zona free mouse blastocysts, binds to a 75-kDa cortical granule protein (p75) present in a subpopulation of mouse cortical granules. The purpose of this study was to identify and characterize p75, examine its distribution in unfertilized oocytes and preimplantation embryos, and investigate its biological role in fertilization.

**Results:**

To identify p75, the protein was immunoprecipitated from ovarian lysates with the ABL2 antibody and analyzed by tandem mass spectrometry (MS/MS). A partial amino acid sequence (VLIGGSFY) was obtained, searched against the NCBI nonredundant database using two independent programs, and matched to mouse peptidylarginine deiminase (PAD). When PAD antibody was used to probe western blots of p75, the antibody detected a single protein band with a molecular weight of 75 kDa, confirming our mass spectrometric identification of p75. Immunohistochemistry demonstrated that PAD was present in the cortical granules of unfertilized oocytes and was released from activated and in vivo fertilized oocytes. After its release, PAD was observed in the perivitelline space, and some PAD remained associated with the oolemma and blastomeres' plasma membranes as a peripheral membrane protein until the blastocyst stage of development. In vitro treatment of 2-cell embryos with the ABL2 antibody or a PAD specific antibody retarded preimplantation development, suggesting that cortical granule PAD plays a role after its release in preimplantation cleavage and early embryonic development.

**Conclusion:**

Our data showed that PAD is present in the cortical granules of mouse oocytes, is released extracellularly during the cortical reaction, and remains associated with the blastomeres' surfaces as a peripheral membrane protein until the blastocyst stage of development. Our in vitro study supports the idea that extracellular PAD functions in preimplantation development.

## Background

Mammalian cortical granules are membrane-bound organelles located in the cortex of unfertilized oocytes [1,2]. Following gamete membrane fusion, cortical granules undergo exocytosis, and some of the released components block polyspermy by modifying the zona pellucida [3-14]. In addition, some cortical granule proteins remain associated with the embryo and appear to regulate embryogenesis, since *in vitro *culture of 2-cell embryos in the presence of antibodies specific to these proteins inhibited embryo cleavage [15-17]. While most cortical granules are released after fertilization, a subpopulation of *Lens culinaris *agglutinin (LCA)-binding cortical granules are released around the cleavage furrow during first polar body extrusion [18]. While the biological significance of this pre-fertilization release is not yet known, it likely plays a role in fertilization since it occurs at a specific time and place and involves a specific population of cortical granules. These prior studies show that mammalian cortical granules are released both before and after fertilization and that their functions are probably more complex than previously realized.

The total number of mammalian cortical granule proteins has been estimated to be between four and fourteen or more [10,19,20]. Several specific proteins have been identified as cortical granule proteins [21]. N-acetylglucosaminidase was detected in exudates of ionophore-activated mouse oocytes using an enzymatic assay and was localized in the cortical granules at the electron microscopic level [13]. Approximately 90% of oocyte N-acetylglucosaminidase was released following *in vivo *fertilization and was shown using competitive inhibitors or anti-N-acetylglucosaminidase antibodies to be responsible for the zona block to polyspermy [13]. Ovoperoxidase was detected in the cortical granules of unfertilized mouse oocytes at the ultrastructural level using the 3.3'-diaminobenzidine (DAB) [7,8]. Following artificial activation, ovoperoxidase was present on the oocyte's surface, in the perivitelline space, and in the zona pellucida. Following fertilization, the enzyme was inferred to harden the zona pellucida, since both peroxidase inhibitors and tyrosine analogs prevented hardening [8]. Calreticulin, an endoplasmic reticulum protein involved in calcium storage, was demonstrated in granules in the cortex of hamster oocytes by indirect immunofluorescence [22]. However, a subsequent study showed that most of the granules containing calreticulin did not label with the lectin LCA, a classical marker for mouse oocyte cortical granules [23]. This lead to the conclusion that calreticulin is localized in a population of granules that is distinct from classical cortical granules.

In addition, several proteins (p32, p56, p62, and p75) have been localized immunocytochemically in cortical granules, but their identities have not yet been established [17,19,20]. p32 was recognized on western blots by a monoclonal antibody (3E10) made against mouse cortical granule exudates and was localized immunohistochemically to cortical granules in germinal vesicle intact and metaphase II stage mouse oocytes [19]. Interestingly, p32 was not detected in 3E10 labeled fertilized oocytes and preimplantation embryos following the cortical reaction. While the function of p32 is not known, treatment of unfertilized oocytes with the 3E10 antibody did not increase polyspermy, indicating that for the experimental conditions used, p32 did not function in blocking polyspermy. The polyclonal antibody ABL_2_, which was made against zona free mouse blastocysts and which immunoprecipitates a 75-kDa protein from mouse oocytes, reacts immunocytochemically with cortical granules [20]. The protein is released following *in vitro *fertilization and artificial activation [20]. In hamster oocytes, a pair of cortical granule proteins designated p56 and p62, was recognized on western blots by the ABL_2 _antibody [16]. These two ABL_2 _specific hamster cortical granule proteins are related to sea urchin hyalin since they are also recognized by the *S. purpuratus *hyalin specific antibody IL2 [17]. p56 and p62 are retained in the perivitelline space and on the oolemma after fertilization. These proteins appear to be involved in early embryogenesis since *in vivo *treatment of 2-cell embryos with IL2 or ABL_2 _antibodies inhibited blastomere cleavage [16,17]. *In vitro *treatment of 2-cell mouse embryos with the ABL_2 _antibody showed similar inhibition of development [15]. Although experimental and immunohistochemical work has been done on these cortical granule proteins, they have not yet been identified biochemically or characterized functionally.

The purpose of this study was to identify the mouse cortical granule protein p75, to characterize its distribution in unfertilized oocytes and preimplantation embryos, and to examine its function in fertilization. To accomplish this, p75 was immunoprecipitated from an ovarian lysate, isolated using SDS-PAGE, then analyzed using tandem mass spectrometry. A partial peptide sequence of the protein was obtained and used to identify p75 as a member of the peptidylarginine deiminase (PAD) family of enzymes that catalyze the conversion of arginine to citrulline [24].

## Materials and methods

### Chemicals and Supplies

Chemicals used to make all media, polyvinylpyrrolidone (PVP), bovine serum albumin (BSA), pregnant mare's serum gonadotropin (PMSG), human chorionic gonadotropin (hCG), bovine hyaluronidase, protein A-sepharose beads, M16 medium, paraformaldehyde, Triton-X 100, α-D-mannose, N-acetylglucosamine, β-D-galactose, and N-acetylgalactosamine were purchased from Sigma Chemical Company (St. Louis, MO). HEPES buffer, light mineral oil, slides, and coverslips (#1.5) were purchased from Fisher (Tustin, CA). *Lens culinaris *agglutinin (LCA), streptavidin conjugated to Texas Red, and Vectashield mounting medium were purchased from Vector Laboratories (Burlingame, CA). SYTOX orange nucleic acid stain and Alexa-488 conjugated to goat anti-rabbit IgG were obtained from Molecular Probes (Eugene, OR). PAD V (N) antibody was made against recombinant human PAD V and affinity purified on an N-terminal PAD V fragment (1–262) bound column as previously described [25]. ePAD antibody was made against the N-terminal fragment (1–200) of mouse recombinant ePAD [26].

### Animals

NIH Swiss white mice were purchased from Harlan (San Diego, CA). Mice were housed in a University of California at Riverside vivarium with a 14-hour light and 10-hour dark cycle and fed water and Purina rodent chow (Ralston-Purina, St. Louis, MO) ad libitum. Protocols used in this study were approved by the campus Committee on Animal Care.

### Media and Fixatives

For dissection and oocyte collection, Earle's balanced salt solution with 28.18 mM of sodium bicarbonate and 24.98 mM of HEPES free acid (EBSS-H), pH 7.4 supplemented with 0.3% of polyvinylpyrrolidone (EBSS-H/0.3% PVP) was made as previously described [27]. For immunoprecipitation, lysis buffer was made with 150 mM NaCl, 10% NP-40, 0.5% sodium deoxycholate, 0.1% SDS, 50 mM Tris-HCl, pH 7.5, and a protease inhibitor cocktail as previously described [13]. For egg activation, calcium and magnesium free EBSS-H (EBSS^-Ca/Mg^-H) was used as previously described [19]. High salt-containing solution was made by increasing the sodium chloride concentration in EBSS-H/0.3% PVP to 300 mM. For embryo culture, M16 medium was pregassed in 37°C humidified incubator (5% CO_2_, 95% air) overnight before use. For confocal scanning laser microscopy, Dulbecco's phosphate buffered saline (DPBS), pH 7.4 or phosphate buffered saline (PBS), pH 7.4 was used. DPBS was made with 90.9 mM CaCl_2_, 2.68 mM KCl, 1.47 mM KH_2_PO_4_, 0.49 mM MgCl_2_·6H_2_O, 136.89 mM NaCl, and 8.06 mM Na_2_HPO_4_·7H_2_O. PBS was made as described previously [25]. For fixation, 4% paraformaldehyde was made in DPBS, pH 7.4, or in PBS, pH 7.4. Blocking solution was made in DPBS, pH 7.4 supplemented with 7.5 mg/ml glycine and 3 mg/ml BSA immediately prior to use. In some cases, blocking solution was made in PBS. 10 mM citrate buffer pH 7.0 was made with 3.78 g of citric acid and 2.411 g of sodium citrate in 1 L of H_2_O. To remove peripheral ABL_2 _specific antigen following egg activation, high salt-containing EBSS-H/0.3% PVP containing 300 mM NaCl was used. For confocal scanning laser microscopy, labeling solution was made by supplementing DPBS, pH 7.4 with 30 mg/ml BSA (DPBS/3% BSA). For LCA blotting, Tris-buffered saline (TBS), pH 7.6 was used (147 mM NaCl; 20 mM Tris-base)

### Oocyte and Embryo Collection

For epifluorescence microscopy, confocal scanning laser microscopy, and gel electrophoresis, oocytes and preimplantation embryos were collected in EBSS-H/0.3% of PVP at room temperature. To collect germinal vesicle intact oocytes, female mice were injected intraperitoneally with 10 IU of PMSG (Sigma, St Louis, MO). Oocytes were collected 60 hours later from the ovaries and mechanically denuded of their cumulus cells with a thin-bore glass pipette. Unfertilized mature metaphase II oocytes were collected from female mice that were primed with 10 IU of PMSG at 2200 hours on day 1 followed by 10 IU of hCG (Sigma, St. Louis, MO) 46 hours later. For egg activation, oocytes were flushed out from oviducts with collection medium 16 to 18 hours post the hCG injection. To collect *in vivo *fertilized oocytes and preimplantation embryos, female mice were superovulated by intraperitoneal injection of 10 IU of PMSG at 1430 hours on day 1 followed by 10 IU of hCG 46 hours later and then placed in cages containing 2–3 male mice. The following day, fertilized oocytes were collected by flushing the oviduct with collection medium. Only oocytes with two pronuclei were used. Two-cell preimplantation embryos were collected by flushing the oviduct with collection medium 2 days after mating. Four- and eight-cell preimplantation embryos were collected by flushing the oviduct or the uterine horns with collection medium 3 days after the mating. Blastocysts were collected by flushing the uterine horns 4 days after mating.

For mature metaphase II oocytes and *in vivo *fertilized oocytes, cumulus cells were removed by incubating oocytes in collection medium containing 100 IU of hyaluronidase for 5 minutes at room temperature. In some experiments, zonae pellucidae were removed with 0.25% pronase in collection medium.

### Human Peripheral Blood Cell Collection

Human peripheral blood cells were obtained from an informed and consenting healthy donor. Red blood cells were removed by sedimentation with dextran 200,000, and the remaining cells were then subjected to Percoll density-gradient centrifugation. Layers containing granulocytes were collected, and cells were then spread onto glass slides by cytospinning.

### Immunoprecipitation

For immunoprecipitation, all steps were carried out in lysis buffer unless otherwise specified. Ovaries from adult female mice were dissected out in EBSS-H and homogenized on ice. The homogenate was kept on ice for one hour then centrifuged at 30,000 g at 4°C for 30 minutes to remove any insoluble material. The supernatants of ovarian homogenate were saved for immunoprecipitation. Homogenates of other tissues were also prepared as described above. Non-specific binding was reduced by incubation of the extracts with normal rabbit serum at 4°C with constant agitation for 90 minutes. To remove any protein-A and Sepharose bead binding proteins before using ABL_2_, protein A-Sepharose beads were then added and incubated with the extracts at 4°C with constant agitation for 30 minutes. The beads were pelleted by a low-speed centrifugation and supernatant was collected. The clean ovarian extracts were incubated overnight with ABL_2 _at a final concentration of 0.37 mg/ml at 4°C with constant agitation. Fresh protein A-Sepharose beads were added and incubated with the ovarian extracts at 4°C for 90 minutes on the next day. Beads were pelleted by a low-speed centrifugation, and the ovarian extracts were discarded. Beads were rinsed three times for a total of 45 minutes at room temperature, and sample buffer [28] was added.

### Mass Spectrometry

The ABL_2 _immunoprecipitate was excised from the silver stained gel and the sample was sent to W.M. Keck Foundation Biotechnology Resource Laboratory (Yale University, New Haven, CT) for MS/MS identification. The procedures used at the Keck Laboratory are available on the website of the facility . Briefly, in gel trypsin digestion was performed, and protein was eluted with 50% acetonitrile and 0.1% formic acid. The eluted sample was desalted and was then subjected to nanospray MS/MS to obtain amino acid sequences of the tryptic digest.

### Egg Activation

To examine release of PAD from live oocytes using immunofluorescence microscopy, oocytes were activated by incubating them in hyaluronidase for 10–15 minutes. The concentration of hyaluronidase used (approximately 200–250 units) was higher and the length of exposure was longer than is normally used to remove cumulus cells. These conditions of hyaluronidase treatment resulted in activation of most of the oocytes.

To determine if PAD remains associated with the plasma membrane as a peripheral protein after its release from cortical granules, zona free unfertilized metaphase II oocytes were incubated in EBSS^-Ca/Mg^-H supplemented with 0.3% PVP for 15 min at 37°C, and oocytes were artificially activated with 2 μM ionomycin for two minutes at 37°C. Control oocytes were incubated with 0.1% of DMSO for two minutes at 37°C. Activated oocytes were transferred to fresh EBSS-H supplemented with 0.05% PVP droplets under light mineral oil and incubated for 15 minutes at 37°C. Oocytes were then incubated in high salt-containing solution for 2 minutes at room temperature with constant pipetting to remove exocytosed materials from the oocyte surface. Some control oocytes were treated as mentioned above.

### In Vitro Embryo Culture

Zona intact 2-cell preimplantation embryos were collected as described above in the oocyte and embryo collection section. Embryos were cultured in 50 μl of M16 supplemented with 0.02% of gentamycin under mineral oil at 37°C in the incubator (5% CO_2_, 95% air) for three days. The amount of antibody added to the droplet on day one as indicated below: 5 μg for polyclonal rabbit IgG, 1:100 dilution for polyclonal guinea pig IgG, 5 μg for anti-alpha integrin antibody, 5 μg for the antibody ABL_2_, and 1:100 dilution for anti-ePAD antibody. In some experiments, no antibody was added to the droplets. The embryos were checked everyday and total percentage of embryos that reached the blastocyst stage was recorded for each experimental group on day three.

### Confocal Scanning Laser and Epifluorescent Microscopy

All procedures for CSLM were carried out at room temperature under light mineral oil unless otherwise specified. All samples for LCA and ABL_2 _labeling were fixed with 4% paraformaldehyde in DPBS, pH 7.4 for 30 minutes and most samples for PAD labeling were fixed with 4% paraformaldehyde in PBS, pH 7.4 for 30 minutes. Following fixation, samples were washed in blocking solution for a total of 30 minutes and then permeabilized with 0.1% Triton X-100 in blocking solution for 5 minutes. All samples were labeled in labeling solution and each labeling incubation was followed by several washes in fresh labeling solution for a total of 30 minutes. For ABL_2 _labeling, samples were incubated with a 1:300 dilution (40 μg/ml) of ABL_2 _for 30 minutes followed by 30 minutes of incubation in goat anti-rabbit IgG conjugated to Alexa 488 with a 1:300 dilution (6.6 μg/ml). Control samples were incubated with a 1:1000 dilution (28.3 μg/ml) of preimmune rabbit IgG for 30 minutes followed by goat-anti-rabbit Alexa 488. For LCA labeling, samples were incubated with 10 μg/ml of biotinylated LCA for 30 minutes followed by 30 minutes of incubation in 5 μg/ml of Texas Red-streptavidin. Control samples were incubated with 10 μg/ml of LCA that had been preincubated with 100 mM α-methyl-mannopyranoside for 30 minutes followed by 30 minutes of incubation with 5 μg/ml of Texas Red-streptavidin. To double label oocytes or preimplantation embryos, samples were first incubated with ABL_2 _followed by the goat anti-rabbit IgG conjugated to Alexa 488 then incubated with LCA followed by Texas Red-streptavidin as described previously. For PAD labeling, fixed samples were treated with 10 mM citrate buffer for 15 minutes at 95°C, incubated with 2 M Tris-HCl, pH 7.4, for 15 minutes, and then permeabilized with 0.1% Triton X-100 in PBS for 10 minutes. Samples were blocked with 2% normal goat serum and 2% BSA in PBS for 60 minutes and incubated with 1.5 μg/ml of rabbit anti-PAD V overnight. On the following day, samples were incubated in goat anti-rabbit IgG conjugated to Alexa 488 with a 1:300 dilution (6.6 μg/ml) for three hours at room temperature. For LCA and PAD double labeling, samples already labeled with PAD antibody were incubated with 10 μg/ml of LCA for 30 minutes and followed by 30 minutes of incubation with 5 μg/ml of Texas Red-streptavidin on following day. Control samples, non-permeabilized or permeabilized, were incubated with goat anti-rabbit IgG conjugated to Alexa 488 or Texas Red-streptavidin only. All labeled samples were examined using a Zeiss LS 510 confocal scanning laser microscope the next day. Samples were entirely sectioned optically with a space interval determined according to the pinhole setting. For some samples, two-dimensional projections of z-stacks were generated.

To label live unfertilized, activated, or fertilized oocytes with anti-ePAD, samples were incubated at room temperature in M16 culture medium containing anti-ePAD (1:100) for 45 minutes, washed in M16, and incubated 45 minutes at room temperature in M16 containing anti-guinea pig IgG conjugated to Alexa 488 (1:100). Oocytes were then washed and immediately viewed with a Nikon inverted epifluorescence microscope.

For *in vitro *cultured embryos, live embryos that had been incubated in a primary antibody (ABl_2_, anti-ePAD, or anti-integrin) were washed in M16 then incubated in either goat anti-rabbit IgG conjugated to Alexa 488 with a 1:100 dilution (19.8 μg/ml) or goat anti-guinea pig IgG conjugated to FITC with a 1:100 dilution for 1 hour at room temperature. After washing, live samples were examined, and images were taken with a Zeiss epifluorescence microscope.

### Gel Electrophoresis and Lectin Blotting

Protein samples were solubilized with reducing and denaturing Laemmli sample buffer [28] prior to electrophoresis. Samples and biotinylated standards were run in one-dimensional SDS-PAGE Doucet gels (4% stacking/7.5% separating) [29] at 70 V and 140 V respectively and separated proteins were blotted onto nictrocellulose at 100 V for 1 hour [30]. For protein identification by mass spectrometry, the gel was silver stained after electrophoresis as previously described [31]. For lectin blotting, blots were washed in Tris-buffer saline (TBS) for 15 minutes at room temperature and then blocked with 0.5% Tween-20 in Tris-buffer saline (TBT) for 1 hour at room temperature. 1–10 μg/ml of the appropriate biotinylated lectin in TBT was added to the blot for overnight incubation at 4°C with constant agitation. For each control blot, biotinylated lectin was preabsorbed with 100 mM of control sugar for 2 hour at room temperature prior to the overnight incubation. Blots were washed with TBT four times for 60 minutes on the following day and then incubated in a 1:20,000 dilution of HRP-streptavidin in TBT for 40 minutes at room temperature. For PAD immunoblotting, blots were first blocked with 5% nonfat dry milk in PBS with 0.05% Tween 20 (PBT) for 30 minutes at room temperature and then washed with fresh PBT for 15 minutes. Blots were incubated with a 1:4000 dilution of anti-ePAD guinea pig IgG in PBT overnight at 4°C with constant agitation. For controls, all blots were either incubated with a 1:4000 dilution of preimmune guinea pig IgG in PBT or in PBT without antibody added. On the following day, blots were washed for 15 minutes with PBT and incubated with 1:2000 dilution of goat anti-guinea pig IgG conjugated with peroxidase for 2 hours at room temperature. For both lectin and PAD blots, enhanced chemiluminescence (Amersham, Piscataway, NJ) was used to detect bands of interest and band images were captured using Kodak X-Omat autoradiographic films. The molecular weight of protein was calculated using biotinylated standards.

### Statistical Analyses

The percentage of 2-cell preimplantation embryos reaching the blastocyst stage in the presence of different antibodies and the percentage of 2-cell preimplantation embryos reaching the blastocyst stage in the absence of any antibody (control) were analyzed statistically using a one-way analysis of variance (ANOVA) followed by Dunnet's post-hoc test when results of the ANOVA were significant. In both the ANOVA and Dunnet's test, results were considered significant when p ≤ 0.05.

## Results

### The ABL_2 _antibody recognizes a 75-kDa ovarian protein that is present in cortical granules of mouse oocytes

**Figure 1:**
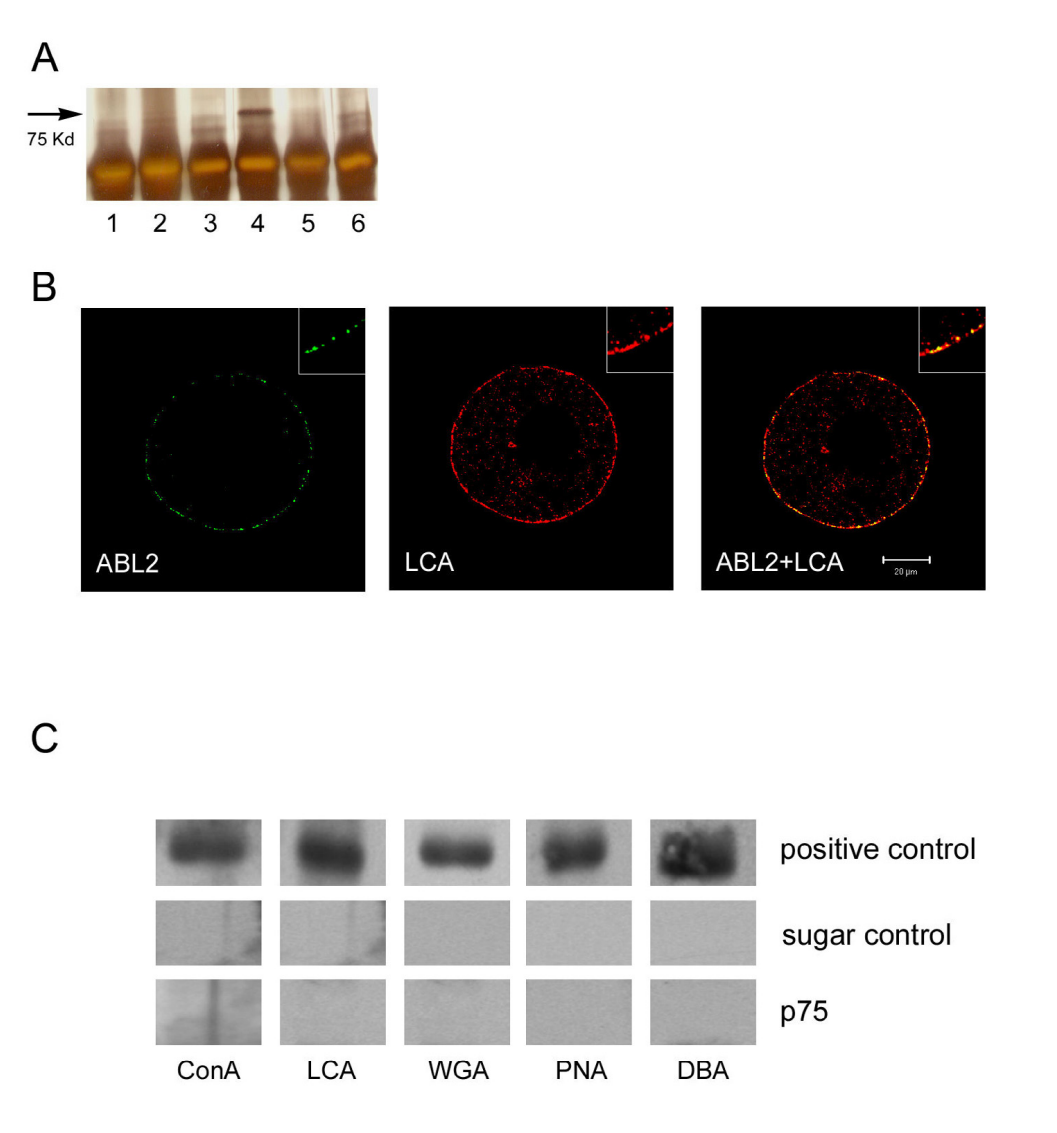
Tissue distribution of the ABL_2 _antigen. (**A**) Silver-stained SDS-PAGE gel loaded with the ABL_2 _immunoprecipitate from mouse brain (lane 1), liver (lane 2), skeletal muscle (lane 3), ovary (lane 4), oviduct (lane 5), and testis (lane 6). The ABL_2 _antibody immunoprecipitated a 75-kDa protein from the ovarian lysate but not from other tissues. Other bands in the gel are from the antibody used for immunoprecipitation. (**B**) Confocal scanning laser micrographs of germinal vesicle intact mouse oocytes double labeled with the lectin LCA (LCA) and the ABL_2 _antibody (ABL_2_). The merged image (LCA + ABL_2_) showed co-localization of LCA and ABL_2 _in some cortical granules. These images were digitally enlarged for better visualization. (**C**) Western blots in which ABL_2 _immunoprecipitate was probed with the lectins ConA, LCA, WGA, PNA, and DBA. Control blots were probed with lectins preabsorbed with the appropriate control sugar. Positive controls (blots with rabbit IgG) were included for each lectin to show that the blotting condition was optimized.

The ABL_2 _antibody precipitates a 75 kDa protein (p75) from mouse oocytes [20]. To determine if other tissues express p75, various mouse tissue extracts were used to perform ABL_2 _immunoprecipitation. p75 was immunoprecipitated from the ovary by ABL_2 _(Fig. 1A, lane 4), but not from brain, liver, muscle, oviduct, or testis (Fig. 1A, lanes 1–3 and lanes 5–6). Both the ABL_2 _antibody (Figs. 1B, ABL_2_) and the lectin Lens culinaris agglutinin (LCA) (Fig. 1B, LCA) labeled granules in the cortex of oocytes. Many granules showed co-localization of the two probes in merged images (Fig. 1B, ABL_2 _/ LCA), demonstrating p75 to be a mouse cortical granule protein. Co-localization of two probes was also observed in pre-translocated cortical granules located in the cytoplasm of germinal vesicle intact oocytes (Figs. 1 and 2 in [18]). Cryosections of mouse ovary did not show ABL_2 _labeling anywhere in the ovary except in the cortical granules (data not shown).

**Figure 2:**
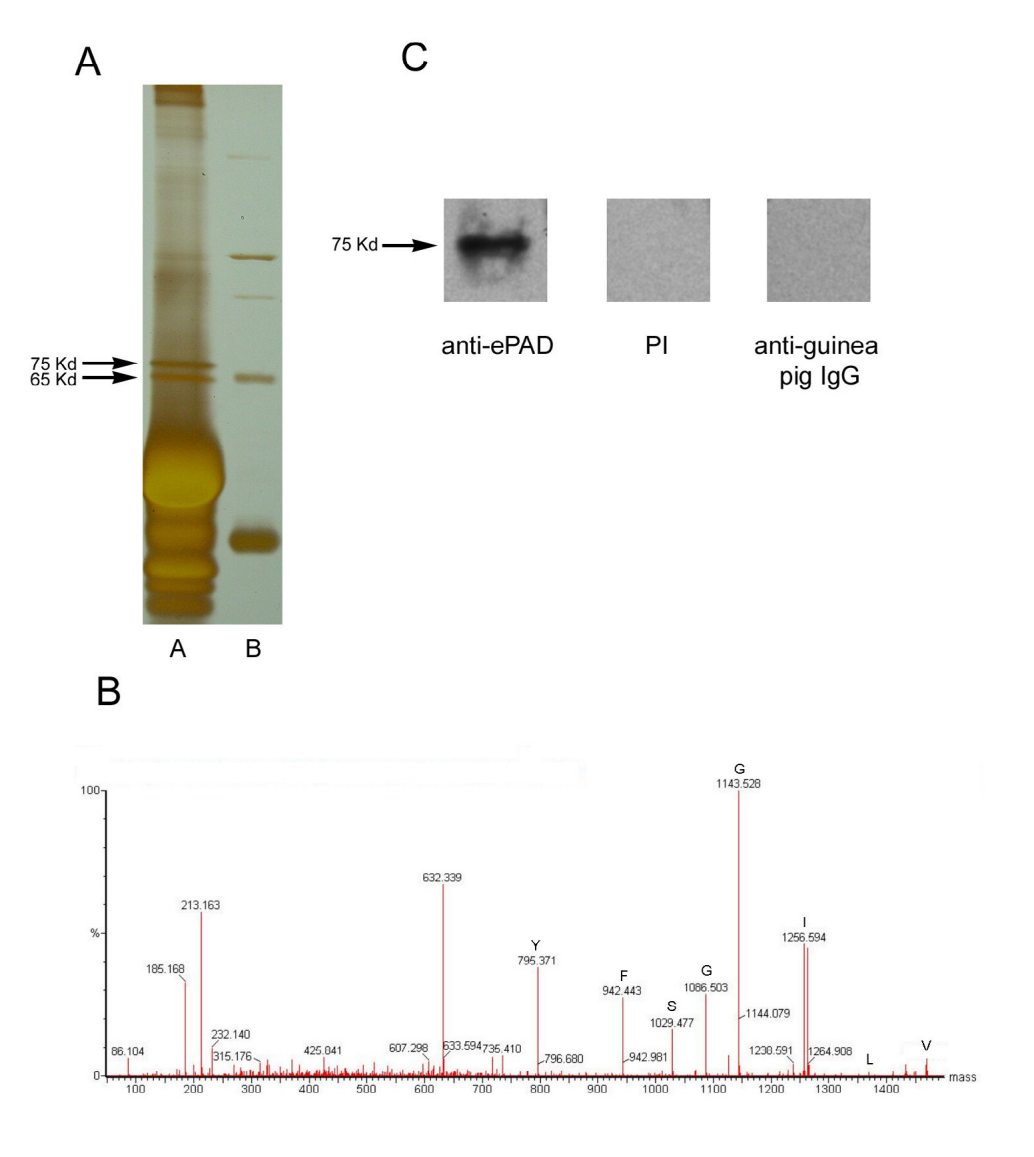
Identification of the ABL_2 _antigen using tandem mass spectrometry. (**A**) Silver-stained SDS-PAGE gel loaded with ABL_2 _immunoprecipitate from mouse ovarian lysate (lane A) and molecular weight standards (lane B). In this experiment, a protein with molecular weight of 65-kDa co-precipitated with p75. (**B**) MS/MS spectrum of a peptide obtained from trypsin-digested p75; the sequence of this peptide was determined to be VLIGGSFY. (**C**) Western blots of an ABL_2 _immunoprecipitate from a mouse ovarian lysate probed with guinea pig anti-ePAD IgG, preimmune guinea pig IgG, or goat anti-guinea pig IgG conjugated to peroxidase only.

Since cortical granule proteins are secreted and most secreted proteins are glycosylated, we performed lectin blotting on immunoprecipitates from ovarian lysates to determine if p75 is glycosylated [32,33]. Blots with p75 were probed with α-D-mannose-specific ConA and LCA, *N*-acetylglucosamine-specific WGA, β-D-galactose-specific PNA, and *N*-acetylgalactosamine-specific DBA. None of these lectins bound to p75 on the blots (Fig. 1C, p75 + lectins), indicating that p75 is probably not glycosylated. Blots with rabbit IgG were used as a positive control to optimize the blotting condition for each lectin and to demonstrate that the assay was working (Fig. 1C, positive control). Control blots probed with lectins preabsorbed with the appropriate sugar under the same blotting conditions did not show binding to rabbit IgG (Fig. 1C, sugar controls), demonstrating the specificity of each lectin.

### Identification of p75 using mass spectrometry

To identify p75, the protein was immunoprecipitated from ovarian lysates with the ABL_2 _antibody and analyzed using mass spectrometry. Generally immunoprecipitation yields a single band of 75 kDa; however, occasionally a second band of 65 kDa is also obtained as shown in Figure 2A. High-energy collision-induced dissociation (CID) spectra of the trypsin-digested of peptides from each protein band was obtained, and partial amino acid sequences of the peptides were deduced. For the 65-kDa band, three peptide sequences were obtained (LVQEVTDFAK/APQVSTPTLVEARAR/LSQTFPNADFAEITK) from the spectra. When sequences were searched separately using BLAST against the NCBI nonredundant database, they all matched serum albumin precursor [GenBank:P07724]. For p75, a CID mass spectrum of the parent peptide ion (at m/z 1468.8^+2^) was obtained and used to deduce the amino acid sequence (Fig. 2B). The spectrum showed a series of peptide ions of decreasing mass generated from the parent peptide. The mass difference between each consecutive peptide ion was used to determine the parent peptide sequence, and a partial amino acid sequence, VLIGGSFY, was then obtained as shown in Figure 2B. The VLIGGSFY sequence matched several mouse peptidylarginine deiminases (PAD) when searched using BLAST against the NCBI nonredundant database. These included a putative mouse PAD type V-like protein [GenBank:XP_144067] predicted by NCBI automated gene predicting algorithm, an egg and embryo abundant PAD [GenBank:AH53724], and a recently characterized mouse oocyte protein, ePAD [GenBank:NP_694746]. Although the egg and embryo abundant PAD (AAH53724) and ePAD (NP_694746) are listed under different entries in the database, they may be the same since their protein sequences are identical except for three amino acids; however, we can not exclude the possibility that they are duplicated genes. In addition, Sonar MS/MS (Genomic Solutions), another software tool designed for mass spectrometric protein identification, was used to search the NCBI nonredundant database. Unlike most database search algorithms that perform protein identification based exclusively on amino acid sequence, Sonar MS/MS includes additional information such as the mass-to-charge (m/z) ratio of the original parent peptide ion to perform identification. This information becomes essential for validating positive protein identification when only a partial amino acid sequence can be obtained from the original parent peptide, as had been the case in this study. The result obtained using Sonar MS/MS showed that the sequence VLIGGSFY was matched to PADs, as had been demonstrated with the BLAST search. To confirm the MS/MS identification of p75, we used an antibody that was made against mouse ePAD [26] to probe blots of the ABL_2 _immunoprecipitate. The ePAD antibody detected a single protein band with a molecular weight of 75 kDa (Fig. 2C, ePAD). No bands were detected when preimmune IgG or goat anti-guinea pig IgG conjugated to peroxidase alone were used (Fig. 2C, PI and anti-guinea pig IgG). These results demonstrate that the p75 immunoprecipitated by ABL_2 _is indeed a PAD and confirm our MS/MS identification of p75.

### Amino acid sequence comparison of different PADs

**Figure 3:**
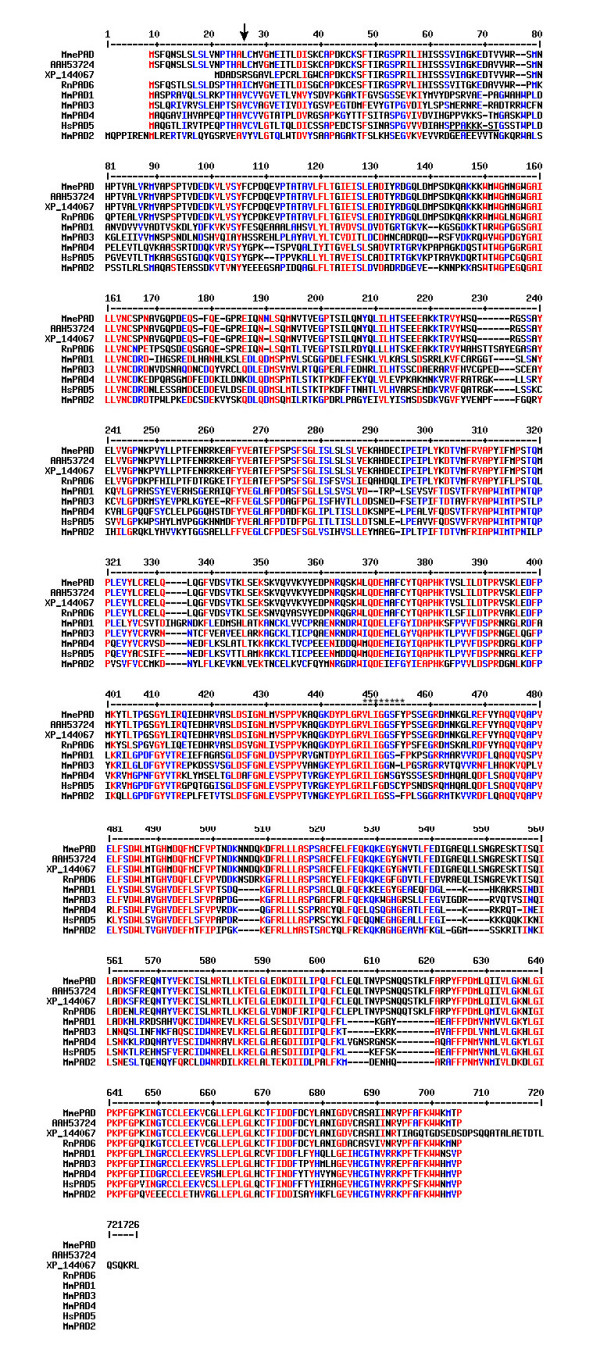
Multiple alignments of mammalian PAD protein sequences. The sequences were aligned using the program MultiAlin available at . The peptide sequence (VLIGGSFY) of p75 obtained from MS/MS analysis was searched against listed PADs and residues that were matched to it are marked (*). The signal peptide cleavage site is marked with an arrow. The monopartite nuclear localization sequence in human PAD V is underlined. High consensus sequences are in red (90% of amino acids are identical or have biochemically similar R-groups) and low consensus sequences are in blue (50% of amino acids are identical or have biochemically similar R-groups). The abbreviations of species are listed as followed: Mm = *M. musculus*; Rn = *R. norvegicus*; Hs = *H. sapiens*. Two putative mouse PAD sequences are referred with their accession numbers (GenBank/NCBI). Accession numbers (GenBank/NCBI) of other PADs are as followed: NP_694746; XP_233601; NP_035189; NP_035190; NP_036519; NP_032838.

Using the MultiAlin program [34], we constructed protein sequence alignments of nine mammalian PAD proteins including all mouse PADs (five characterized mouse PADs: PAD I – IV and ePAD; two uncharacterized mouse PADs, rat PAD VI, and human PAD V [GenBank: NP_035189, NP_032838, NP_035190, AAH53724, XP_144067, NP_694746. XP_233601, NP_036519] (Fig. 3). Sequence residues that are in high consensus are shown in red and sequence residues that are in low consensus are shown in blue. Gaps (-) are introduced for optimal alignment. The multiple alignments of the nine mammalian PADs show that approximately 40% – 50% of the amino acid sequences in these PADs are identical, indicating strong homologies among members of this family. Two predictive algorithms (SignalP V2.0 and TargetP V1.0) [35-37] were used to determine that a putative signal peptide and a cleavage site exist in ePAD and AAH53724 (an egg and embryo abundant peptidylarginine deiminase), indicating they are likely secreted proteins (Fig. 3 arrow). Human PAD V has a monopartite nuclear localization sequence motif [25], and it is the only type of PAD that has been localized to the nuclei of cells (Fig. 3 underline). Only ePAD, AAH53724 (an egg and embryo abundant peptidylarginine deiminase), and XP_144067 (peptidylarginine deiminase type V-like protein) have residues that exactly match the VLIGGSFY sequence (Fig. 3 asterisks). Interestingly, rat PAD VI also has a sequence match to the peptide VLIGGSFY obtained from p75 MS/MS analysis except for the first residue V (Fig. 3 asterisks). PAD I is derived from a gene predictive program, and its sequence is 80% identical to that of ePAD or AAH53724 (an egg and embryo abundant peptidylarginine deiminase).

### Mouse cortical granules contain PAD

**Figure 4:**
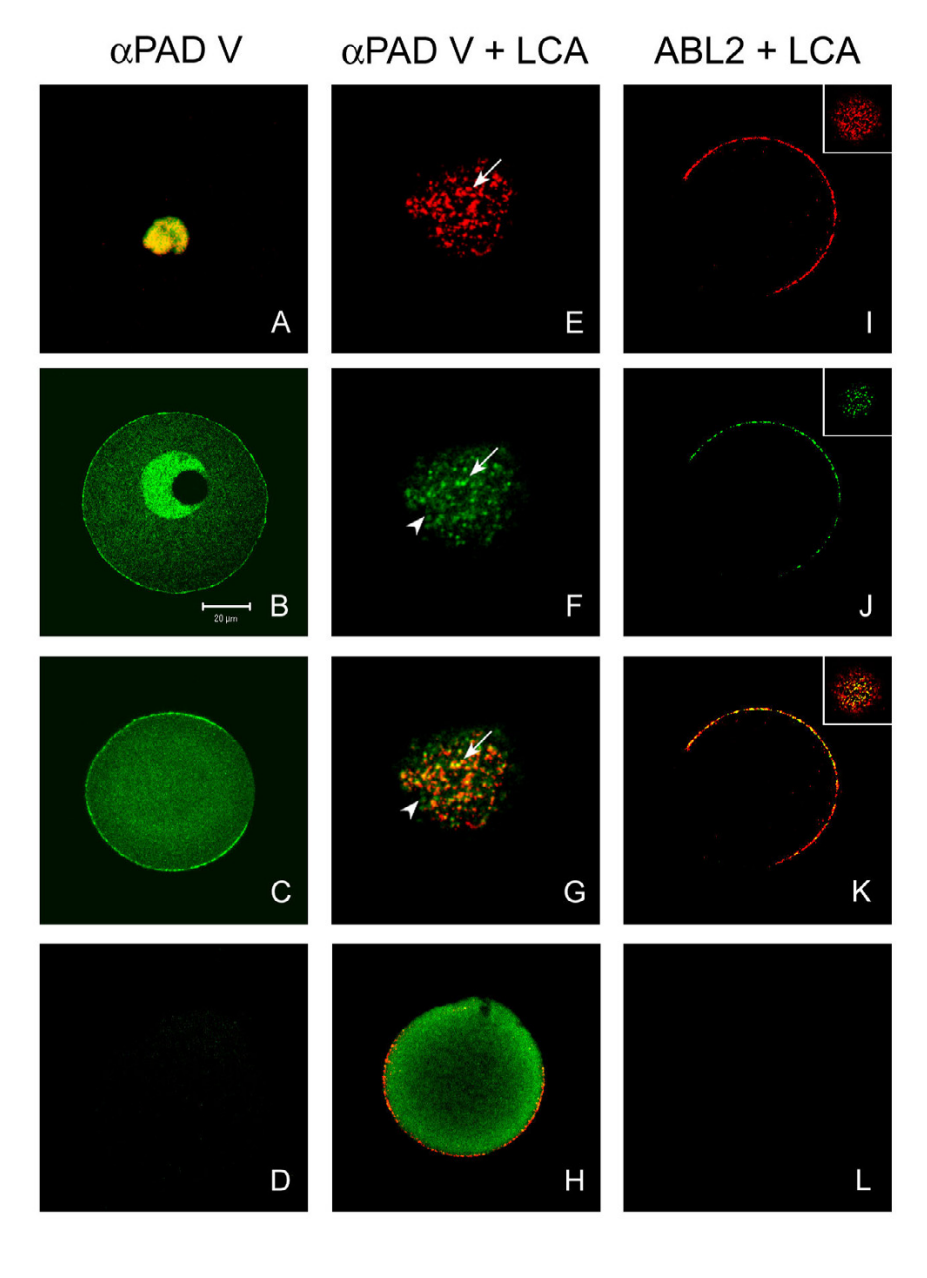
Confocal scanning laser micrographs of (**A**) human blood cells and (**B – D**) *in vivo *matured mouse oocytes labeled with anti-PAD V (N), (**E – H**) *in vivo *matured mouse oocytes double labeled with anti-PAD V (N) and LCA, and (**I – L**) double labeled with ABL_2 _and LCA. All anti-PAD V labeling is shown in green, except in **A **where it is red. DNA stain in **A **is green. ABL_2 _labeling is green and LCA labeling is red in all figures. (**A**) Cytospin preparations of the granulocyte fraction were stained with anti-PAD V (N), and their nuclei were stained with SYTOX green nucleic acid stain. The merged image shows nuclear localization of PAD (yellow) in a human granulocyte. (**B**, **C**) Germinal vesicle intact mouse oocytes and metaphase II oocytes were labeled with anti-PAD V (N), (**D**) Metaphase II mouse oocyte did not show labeling with goat anti-rabbit IgG conjugated to Alexa 488 alone. (**E**, **F**) Polar sections of germinal vesicle intact mouse oocytes double labeled with LCA (red) and anti-PAD V (N) (green). These images were digitally enlarged 2× for better visualization. (**G**) Merged image of both LCA and anti-PAD V (N) showed co-localization (yellow) of labels. (**H**) Merged image of equatorial section of metaphase II mouse oocytes double labeled with anti-PAD V (N) and LCA showing co-localization. (**I**, **J**) Metaphase II oocytes double labeled with LCA (red) and ABL_2 _(green). (K) Merged image of both LCA and ABL_2 _showed co-localization (yellow). The inserts of I, J, and K showed the polar view of the oocyte. (**L**) Control oocytes were not labeled with LCA pre-absorbed with α-D-methyl-mannopyranoside. All samples were imaged at same magnification and the scale bar applies to all figures.

To ascertain if mouse cortical granules contain PAD, antibodies made against mouse ePAD and human recombinant PAD V (anti-PAD V (N)) were used to label *in vivo *matured germinal vesicle intact and metaphase II mouse oocytes. The ePAD antibody had been used previously [26] and showed strong labeling in the cortex. When we adjusted labeling conditions to optimize cortical labeling, both granular and cytoplasmic labeling were observed in the cortex with anti-ePAD; however, the high level of cytoplasmic labeling made it difficult to resolve individual granules and to demonstrate co-localization with LCA, a cortical granule binding lectin (not shown). Therefore the antibody to human PAD-V, which gave a cleaner signal in the cortex, was also used to localize PAD in cortical granules (Fig. 4).

Human peripheral blood cells were first used as a positive control and to optimize labeling conditions with anti-human PAD-V. The antibody labeled only the granulocytes (neutrophils and eosinophils), and labeling was localized to the nuclei of the cells (Fig. 4A), as reported previously [25]. When germinal vesicle intact oocytes were then labeled, immunoreactivity was localized in the nucleus and also in granules in the cortex (Fig. 4B). In metaphase II oocytes, the antibody labeled granules in the cortex; except in the area of the cortical granule free domain which was devoid of PAD labeling (Fig. 4C). In the metaphase II oocytes, the nuclear envelope had broken down, and thus there was no nuclear staining; however, the cytoplasm of metaphase II oocytes was more intensely labeled than that of germinal vesicle intact oocytes, suggesting that nuclear PAD was now dispersed in the cytoplasm (Figs. 4B, C). These results demonstrate that PAD is present in the cortical granules, nucleus, and cytoplasm of unfertilized mouse oocytes. Control oocytes were not labeled with goat anti-rabbit IgG conjugated to Alexa 488 alone (Fig. 4D).

To confirm that anti-PAD V (N) is labeling cortical granules in the oocyte's cortex and that PAD is present in these granules, anti-PAD V (N) and LCA were used to double label germinal vesicle intact and metaphase II oocytes, and their labeling pattern was compared to that of ABL_2 _and LCA double labeled oocytes. Both anti-PAD V (N) and LCA labeled granules (arrow) in the cortex of germinal vesicle intact oocytes (Figs. 4E, F). When images of both probes were merged, many granules appeared orange or yellow indicating co-localization of these probes (Fig. 4G), and similar co-localization of granules was also observed when metaphase II oocytes were used (Fig. 4H). In the metaphase II oocytes, an area devoid of signal corresponding to the cortical granule free domain was observed (Fig. 4H), and this domain was not labeled by either anti-PAD V (N) or LCA. When ABL_2 _and LCA were used to double label metaphase II oocytes, both probes labeled the granules in the cortex and showed co-localization of granules (Figs. 4I–K), as had been observed with anti-PAD V (N) and LCA. Besides the granules in the cortex, anti-PAD V (N) also labeled cytoplasm near the cortical granules; however, this labeling is diffuse and less granular than the cortical granule labeling. This diffuse cytoplasmic labeling did not co-localize with LCA labeling (Figs. 4F, G, arrowhead). Control oocytes labeled with LCA pre-absorbed with α-D-methyl-mannopyranoside showed no labeling (Fig. 4L). Taken together, these results demonstrate that antibodies to PAD label cortical granules of mouse oocytes as had been observed with the ABL_2 _antibody and that PAD (ABL_2 _antigen, p75) is present in the cortical granules of mouse oocytes.

### Localization of PAD (p75) after artificial activation and fertilization

**Figure 5:**
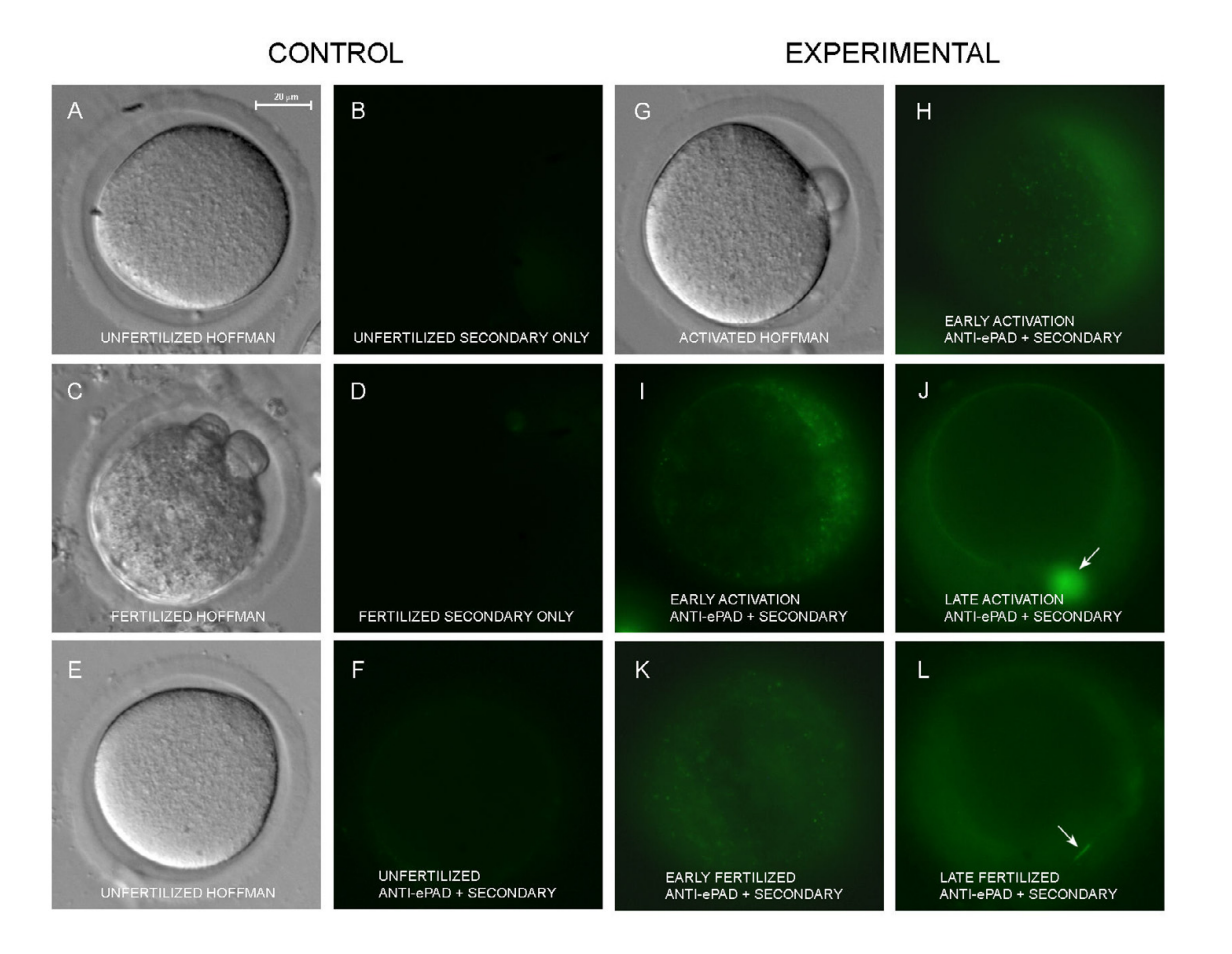
Live, non-permeabilized activated and *in vivo *fertilized oocytes showing release of PAD from the cortical granules. **A **and **B **are the same unfertilized oocyte viewed with Hoffman optics (**A**) or epifluorescence microscopy (**B**) after labeling with the secondary antibody only. No non-specific labeling is observed in **B**. **C **and **D **are the same fertilized oocyte viewed with Hoffman optics (**C**) or fluorescence microscopy (**D**) after labeling with secondary antibody only. No non-specific labeling is observed after fertilization. **E **and **F **are the same unfertilized oocyte viewed with Hoffman microscopy (**E**) or epifluorescence microscopy (**F**) after labeling with both the anti-ePAD and the secondary antibody. No labeling is observed around the unfertilized oocyte (**F**). **G**, **H**, and **I **are the same early activated oocyte viewed with Hoffman optics (**G**) or after labeling with both anti-ePAD and secondary antibody (**H, I**). **H **is focused close to the surface of the oocyte and shows numerous small labeled granules in the perivitelline space. **I **is focused near the equator of the oocyte and shows larger dispersing granules and diffuse label in the perivitelline space. **J **shows a different oocyte at a later state of activation. Label has adhered to the surface of the oocyte and the polar body (arrow). Diffuse label is present in the perivitelline space. **K **and **L **are double labeled fertilized oocytes recovered from oviducts of naturally mated females. **K **shows an earlier stage after fertilization in which some label in the perivitelline space is still granular. **L **shows a later stage after fertilization in which label is diffuse in the perivitelline space. In **K**, a sperm tail in the perivitelline space has apparently absorbed PAD (arrow).

To demonstrate that PAD is released from cortical granules when they undergo exocytosis, unfertilized, hyaluronidase activated, and *in vivo *fertilized oocytes were compared using immunofluorescence microscopy (Fig. 5). All oocytes were labeled live (non-permeabilized) with the primary and secondary antibody and were imaged using an inverted epifluorescent microscope to minimize damage to the living oocytes. Since only extracellular PAD was imaged in this experiment, anti-ePAD was used, and cortical cytoplasmic labeling did not interfere with interpretation of the images, as had occurred when oocytes were permeabilized and imaged with confocal microscopy (see previous section). Secondary antibody alone did not label unfertilized or fertilized oocytes (Figs. 5A–B, C–D). Unfertilized live oocytes did not show extracellular fluorescence when labeled with both anti-ePAD and the secondary antibody (Figs 5E–F), Oocytes caught in various stages of activation showed distinct patterns of extracellular labeling with anti-ePAD (Figs 5G–J). In early stages of activation, numerous extracellular granules were labeled in the perivitelline space (Figs. 5H–I). Many of these granules were the size of cortical granules suggesting they were recently exocytosed (Fig 5H). Other granules had begun to disperse and were larger in diameter (Fig 5I). At later times after activation, granular content had dispersed completely within the perivitelline space, and some labeling appeared associated with the oolemma (Fig 5J). Similar to activated oocytes, fertilized oocytes that were recovered from oviducts of mated females had labeled granules in the perivitelline space (Fig 5K). At later stages, the contents of the granules had dispersed to fill the perivitelline space (Fig. 5L).

### Localization of PAD versus other cortical granule components during preimplantation development

**Figure 6:**
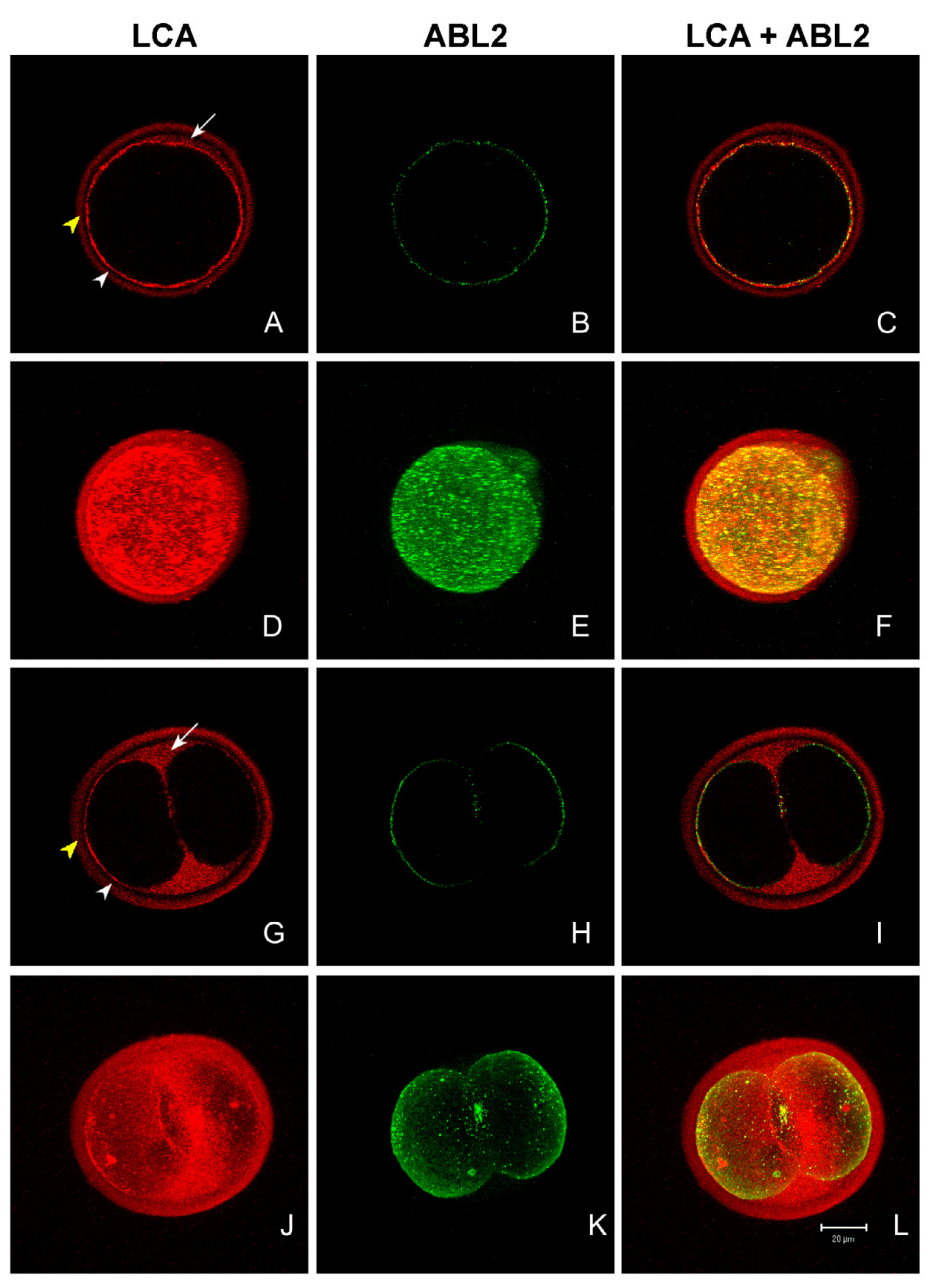
Confocal scanning laser micrographs comparing distribution of PAD and LCA-binding cortical granule components in *in vivo *fertilized oocytes and *in vivo matured *2-cell embryos. Optical sections (**A – C **and **G – I**) and two-dimensional projections of z-series (**D – F **and **J – L**) of zona intact fertilized oocytes (**A – F**) and zona intact 2-cell embryos (**G – L**) labeled with LCA (red) and ABL_2 _antibody (green). **A**, **D**, **G**, and **J **show the LCA labeling on the surface of the oocyte (white arrowhead), in the perivitelline space (arrow), and in the zona pellucida (yellow arrowhead). **B**, **E**, **H**, and **K **show the ABL_2 _labeling. **C**, **F**, **I**, and **L **are merged confocal images and two-dimensional projections showing both LCA and ABL_2 _labeling.

To follow the fate of secreted PAD during preimplantation development and to compare the fate of secreted PAD to glycosylated cortical granule components, fixed *in vivo *fertilized oocytes and *in vivo *matured preimplantation embryos were double labeled with the ABL_2 _antibody and LCA (Fig. 6). LCA would be expected to localize glycosylated cortical granule components, while ABL_2 _would localize secreted PAD. ABL_2 _was used to localize PAD in this experiment since anti-ePAD images were difficult to interpret using fixed permeabilized samples and since the method used for anti-PAD V labeling removed the zona which precluded tracing secreted material into the perivitelline space or zona.

After fertilization, the released LCA-binding cortical granule components were present mainly on the surface of oocytes (Fig. 6A white arrowhead; Fig. 6D) and in the zona pellucida (Fig. 6A yellow arrowhead; Fig. 6D). Less intense LCA labeling was observed in the perivitelline space (Fig. 6A arrow; Fig. 6D). In contrast, ABL_2 _labeling was detected on the surface of fertilized oocytes (Fig. 6B, E) and in the perivitelline space (Fig. 6E), but not in the zona pellucida of the fertilized oocytes (Fig. 6B, E), in agreement with Figure 4. Fixed non-permeablized fertilized oocytes showed the same LCA and ABL_2 _labeling patterns (data not shown).

In merged images of double-labeled fertilized oocytes, much of the LCA and ABL_2 _labeling on the oocyte's surface was co-localized (Fig. 5C). In two-dimensional projections of z-series of the double labeled fertilized oocytes that contained pronuclei and two polar bodies, the LCA and ABL_2 _labeling present on the surface of the oocytes was granular, and hence similar in appearance to the cortical granules of unfertilized oocytes (Figs. 6D–F). Many of these extracellular granules were larger than the cortical granules in unfertilized oocytes, indicating they had dispersed slightly by this stage, as seen previously in unfixed activated and fertilized oocytes (Figs. 5H–J).

At the 2-cell stage, some of the LCA-binding cortical granule components (Fig. 6G white arrowhead) and all of the ABL_2 _antigen (Fig. 6H) remained associated with the blastomeres' plasma membranes with some labeling observed between the blastomeres (Figs. 6G–I). Two-dimensional projections of series of z-stacks revealed that LCA and ABL_2 _labeling on the blastomeres' surfaces was diffuse, not granular, at this stage (Figs. 6J, K, L). In merged images, LCA and ABL_2 _labeling showed less co-localization on the 2-cell preimplantation embryos' surface than on the fertilized oocytes' surface (compare Fig. 6C and Fig. 6I). Unlike ABL_2_, LCA-binding cortical granule components were evenly dispersed in the perivitelline space (Figs. 6G arrow, I, J, L) and in the zona pellucida (Figs. 6G yellow arrowhead; I, J, L).

**Figure 7:**
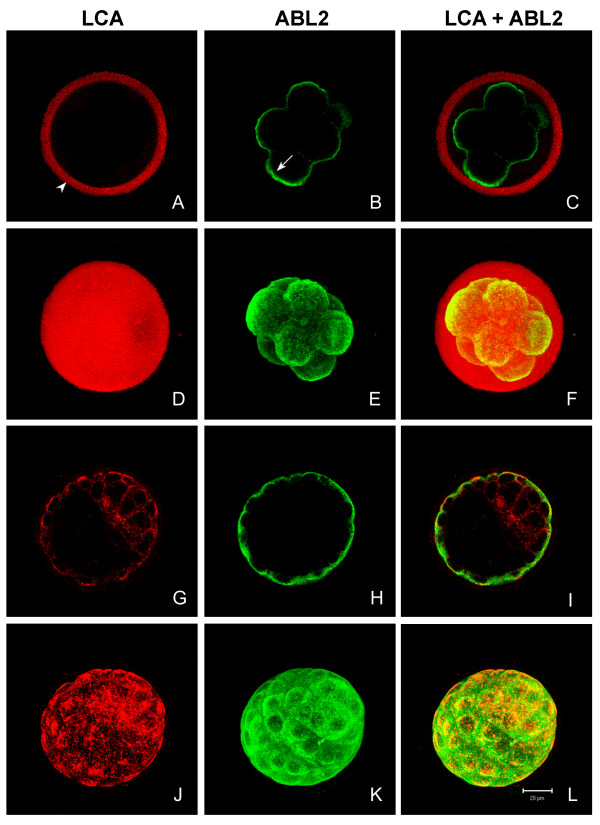
Confocal scanning laser micrographs comparing distribution of PAD and LCA-binding cortical granule components in *in vivo *matured 8-cell embryos and blastocysts. Optical sections (**A – C **and **G – I**) and two-dimensional projections of z-series (**D – F **and **J – L**) of zona intact 8-cell embryos (**A – F**) and blastocysts (**G – L**) labeled with LCA (red) and ABL_2 _(green). **A **and **D **show the LCA labeling in the zona pellucida (arrowhead). **B **and **E **show ABL_2 _labeling on the plasma membranes and in the subcortical region (arrow) of blastomeres. **G **and **J **show LCA labeling of the trophoblast cells and the inner cell mass cells. **H **and **K **show ABL_2 _labeling of the trophoblast cells. **C**, **F**, **I**, and **L **are merged confocal images and two-dimensional projections showing both LCA and ABL_2 _labeling.

At the 8-cell stage, LCA-binding components were found in the zona pellucida (Figs. 7A, C, D, F), but not in the perivitelline space or on the blastomeres' plasma membranes (Figs. 7A, C). ABL_2 _labeling was still associated only with the blastomeres' plasma membranes, where it appeared diffuse, not granular (Figs. 7B, C, E). Lack of co-localization of LCA and ABL2 on the blastomeres surface at this time supports our data (Fig. 1C) that the ABL_2 _antigen (PAD) is not glycosylated. At the 8 cell stage, ABL_2 _also labeled the subcortical region of blastomeres (Fig. 6B arrow), consistent with previous reports at this stage [15,38].

At the early blastocyst stage, LCA labeling was present on the surface of the trophoblast and the inner cell mass cells (Figs 7G, I, J, L), while ABL_2 _labeling was found only on the surface of the trophoblast cells (Figs. 7H, I, K, L). The LCA labeling on the trophoblast was patchy (Figs. 7G, J), whereas the ABL_2 _labeling was evenly dispersed (Figs. 7H, K). It is probable that the LCA staining observed at this stage did not exclusively represent LCA-binding cortical granule proteins but rather newly synthesized surface proteins. Neither LCA nor ABL_2 _labeling was detected in the perivitelline space or in the zona pellucida (Figs. 7G–L). Control fertilized oocytes and preimplantation embryos were not labeled by preimmune IgG, LCA pretreated with α-methyl-mannopyranoside, or Texas Red-streptavidin alone (data not shown).

**Figure 8:**
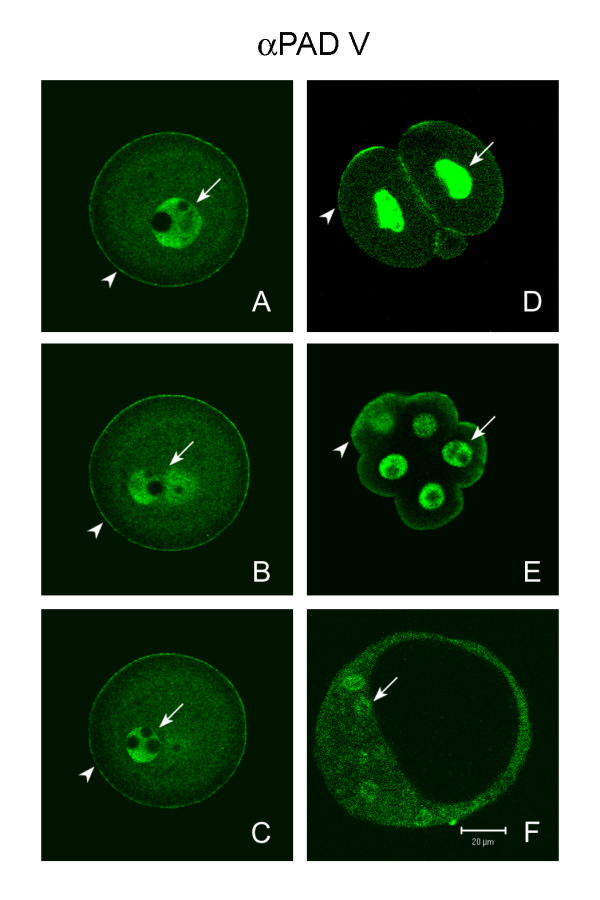
Confocal scanning laser micrographs showing distribution of PAD in *in vivo *fertilized oocytes and *in vivo matured *pre-implantation embryos labeled with anti-PAD V (N). (**A – C**) Three different focal sections of a zona free fertilized oocyte showing PAD labeling on the oocyte's surface (arrowhead) and in both pronuclei (arrows). (**D**, **E**) A 2-cell embryo and an 8-cell embryo showed PAD labeling on the blastomeres' surfaces (arrowhead) and in their nuclei (arrows). (**F**) A blastocyst showing that anti-PAD V (N) labeled the trophoblast cells, and inner cell mass cells, and nuclei of inner cell mass cells (arrow).

The previous experiment demonstrated that secreted PAD was in the perivitelline space and on the oolemma immediately after fertilization, but by the 2 cell stage was found only on the oolemma. In contrast, other cortical granule components that label with LCA passed into the perivitelline space and zona pellucida immediately after fertilization and were not found on the blastomeres' plasma membranes by the 8 cell stage. To confirm the localization of PAD on plasma membranes, *in vivo *matured preimplantation embryos were also labeled with anti-PAD V. In addition to labeling secreted PAD, anti-PAD V would also be expected to label nuclear and cytoplasmic forms of PAD as shown in Figure 4. Anti-PAD V was used in this experiment since it gave cleaner, more interpretable images, especially near the surface of oocytes, than anti-ePAD. Anti-PAD V (N) labeled the surface of fertilized oocytes (Figs. 8A–C arrowheads). The zona and hence the perivitelline space were lost from these oocytes during immunolabeling, therefore localization in the perivitelline space could not be confirmed with this antibody. After fertilization, PAD labeling was evenly distributed and continuous on the surface of oocytes, in contrast to unfertilized oocytes in which an area devoid of PAD labeling (cortical granule free domain) was observed above the spindle (Fig. 4C). After fertilization, both the pronuclei (arrow) and the cytoplasm were also labeled which would be expected for the anti-PAD V (N) antibody (Figs. 8A–C).

At the 2-cell stage, PAD labeling still remained on the blastomeres' plasma membranes, and some immunoactivity was found between the two blastomeres (Fig. 8D arrowhead) as had been observed with the ABL_2 _antibody (Fig. 6H). In addition, both the nuclei (Fig. 8D arrow) and to a lesser extent the cytoplasm of two blastomeres were stained by anti-PAD V (N). At the 8-cell stage, anti-PAD V (N) labeling was still associated with the blastomeres' plasma membranes, and the label was diffuse around the blastomeres surface (Fig. 7E, arrowhead). In addition, PAD labeling was also found deeper in the cortical cytoplasm (Fig. 8E), as was observed with the ABl_2 _antibody at the 8 cell stage (Fig. 7H) [39]. Anti-PAD V (N) also strongly labeled the nuclei (arrow) and weakly labeled the cytoplasm of each blastomere at the 8 cell stage (Fig. 8E). Finally, in blastocysts, anti-PAD V (N) labeled the cytoplasm of both trophoblast and inner cell mass cells with equal intensity (Fig. 8F). This antibody also labeled nuclei of inner cell mass cells (Fig. 8F arrow) and trophoblast cells (not shown in this focal section) at this stage. It was not possible to determine if the surface of the blastocyst was labeled by anti-PAD-V at this stage due to cytoplasmic labeling that extended to the periphery of the cells.

**Figure 9:**
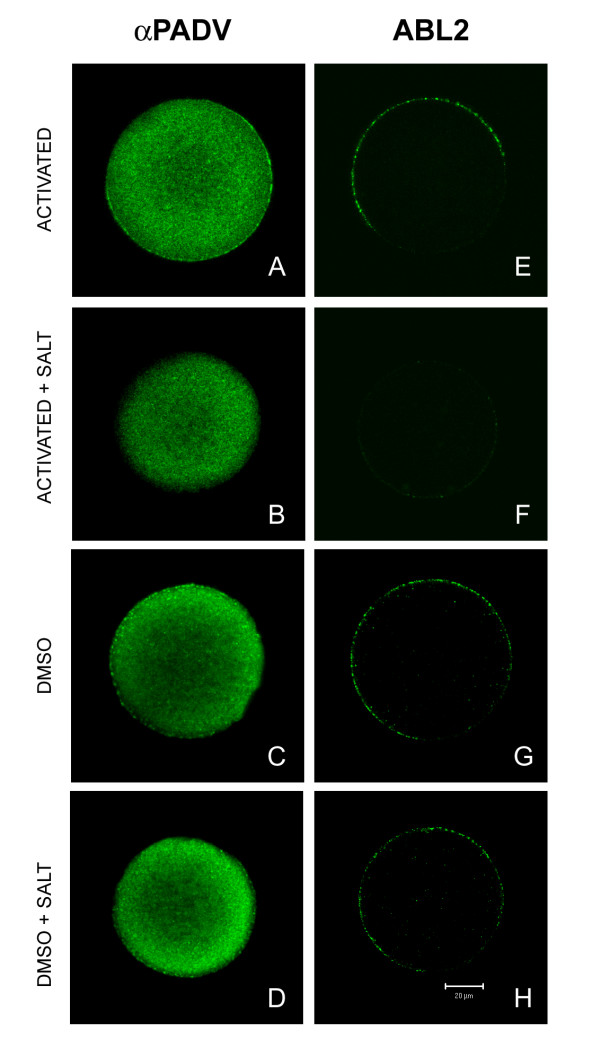
Confocal scanning laser micrographs of *in vivo matured *metaphase II oocytes labeled with either anti-PAD V (N) (**A – D**) or ABL_2 _(**E – H**). showing that PAD is a peripheral membrane protein. A and E are artificially activated oocytes showing released PAD and ABL_2 _antigen (p75) on the oocyte's surface. **B **and **F **are artificially activated oocytes treated with a high salt solution that removed PAD and p75 from oocytes' surfaces. **C **and **G **are DMSO control oocytes showing PAD and ABL_2 _antigen in cortical granules. **D **and **H **are DMSO control oocytes treated with a high salt solution confirming that PAD and ABL_2 _antigen were still detected in cortical granules.

Figures 5, 6, 7, 8, above demonstrate that PAD is released from cortical granules following fertilization and that at least some PAD remains associated with the oocyte and blastomeres' surfaces during preimplantation development. Since PAD was observed in the perivitelline space of living activated and fertilized oocytes, it is possible that some of this secreted PAD binds back to the oolemma as a peripheral membrane protein. Alternatively, the PAD associated with the oolemma may represent a different isoform of PAD that is in fact an integral membrane protein. To distinguish between these two possibilities, we treated artificially activated oocytes with high salt-containing solution. If PAD is peripherally associated with the oolemma following exocytosis, high salt-containing solution should remove it from the oocyte's surface. If PAD is an integral membrane protein, it should remain on the surface following this treatment. Both anti-PAD V (N) and ABL_2 _labeled the surface of artificially activated oocytes (Figs 9A, E), and the labeling was removed from the surface when activated oocytes were treated with high salt-containing solution (Figs. 9B, F). Control non-activated oocytes showed PAD and ABL_2 _labeling in the cortical granules (Figs. 9C, G), and treatment of non-activated oocytes with high salt-containing solution did not modify this labeling (Figs. 9D, H). The control was conducted to ensure that the PAD and ABL_2 _labeling were removed from the activated oocyte's surface by high salt treatment and not by DMSO that was used to dissolve ionomycin for artificial activation. These results show that PAD on the oolemma behaves as a peripheral membrane protein after it is released from cortical granules by artificial activation.

### Role of PAD in preimplantation embryonic development

**Figure 10:**
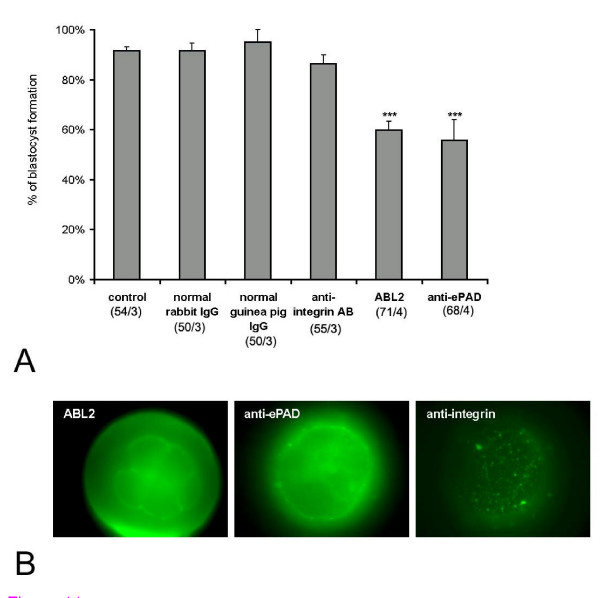
(**A**) Effects of ePAD antibody and ABL_2 _antibody on preimplantation development. **A **shows the percentage of blastocyst formation in the presence of different antibodies. The number of experiments and the total number of oocytes are shown in the figure for each experimental group. (**B**) Epifluorescent micrographs of *in vitro *matured live 8-cell embryos treated with ABL_2 _antibody, anti-ePAD, and β1 integrin antibody followed by the appropriate secondary antibody. In each case label is present on the blastomeres' surfaces. (***) *P *< 0.01.

Previously, the ABL_2 _antibody was shown to inhibit hamster and mouse preimplantation embryonic development [16]. To determine if antibodies to PAD show a similar inhibitory effect on mouse preimplantation development, *in vivo *matured zona intact 2-cell embryos were incubated *in vitro *in the presence of PAD or control antibodies, and the number of blastocysts was counted on day 3 (Fig. 10A). When control embryos were cultured in the absence of any antibodies, most embryos (90%) showed normal development to the blastocyst stage. Both ABL_2 _and anti-ePAD inhibited development of 2-cell embryos. In the presence of ABL_2 _and ePAD antibodies, only 60% or 55% respectively of 2-cell embryos reached the blastocyst stage. Higher concentrations of anti-ePAD produced significantly greater inhibition of development with only 22% of the 2 cells stage becoming blastocysts (not shown). In the ABL_2 _and anti-ePAD treatment groups, most embryos that did not develop to the blastocyst stage were either in the 8-cell or the morula stage on day 3 (data not shown), indicating that cleavage divisions were inhibited in the presence of ABL_2 _and PAD antibodies. *In vitro *treatment of 2-cell embryos with preimmune rabbit IgG, preimmune guinea pig IgG, and function-blocking rabbit anti-β1 integrin IgG did not significantly affect embryo development (Fig. 10A) demonstrating that the results seen with the ABL_2 _and PAD antibodies were specific and not simply due to IgG binding to the cell surface. To show that the antibodies to PAD and β1 integrin bound to the blastomeres' surfaces, live 8-cell embryos treated with each antibody were subsequently incubated with anti-rabbit or anti-guinea pig IgG conjugated to FITC. Epifluorescent micrographs showed that all of these antibodies bound to the blastomeres' surfaces (Fig. 10B). These results demonstrate that cleavage divisions and blastocyst formation were significantly inhibited by a PAD specific antibody.

## Discussion

In the present study, a mouse cortical granule protein, p75, was immunoprecipitated from ovarian lysate, microsequenced by tandem mass spectrometry, and identified as peptidylarginine deiminase (PAD) using two independent software tools (BLAST and Sonar MS/MS). PAD was secreted from cortical granules following artificial activation or fertilization. Secreted PAD was present in the perivitelline space and on the oolemma of freshly activated or fertilized oocytes. Unlike LCA-binding cortical components which diffused into the zona pellucida, PAD remained attached to the plasma membrane of blastomeres at later times in preimplantation development. PAD on the plasma membrane of activated oocytes could be removed by high salt treatment indicating that it was a peripheral membrane protein. PAD appears to be a non-glycosylated secretory protein as it did not bind any tested lectin in blots and it did not bind LCA in confocal sections of 8 cell embryos. *In vitro *experiments with antibodies to PAD suggest that cortical granule PAD plays a role, after its release at fertilization, in cleavage and early development.

Various lines of evidence support the conclusion that PAD is a cortical granule protein equivalent to p75, the antigen immunoprecipitated by the ABL_2 _antibody. First, a PAD specific antibody (anti-ePAD) recognized p75 on Western blots. Secondly, oocyte PAD and p75 immunoprecipitated with the ABL2 antibody from mouse oocytes have the same molecular weights (75 kDa) and similar isoelectric points (pI) on 2-dimensional gels (5 to 5.5 for PAD and 4.9 to 5.3 for p75) [20,26]. Following the cortical reaction, both p75 and PAD remained associated with the plasma membrane during early preimplantation embryonic development. In the embryo culture experiments, antibodies to both p75 and PAD inhibited cleavage and preimplantation development. Finally, PAD and the lectin LCA were co-localized in some, but not all, cortical granules in agreement with our earlier observation using the ABL_2 _antibody [18]. When taken together, the above evidence supports the conclusion that p75 is PAD, which is localized in mouse cortical granules.

Cortical granule PAD is the first member of the PAD family that has been reported to be secreted. While most secreted proteins are glycosylated, our lectin blots suggest that the cortical granule PAD is not, a conclusion also supported by the observation that the molecular weight of cortical granule PAD immunoprecipitated from mouse ovaries (75 kDa) is similar to the molecular weight of PAD computed from its amino acid sequence (76.7 kDa). Moreover, LCA and ABl_2 _did not co-localize at the 8 cell stage of preimplantation development, further indicating that LCA does not bind to PAD. Several other non-glycosylated proteins, such as chemokines, albumin, and transcobalamin II, are also secreted [40-42].

PADs are a family of calcium dependent enzymes that catalyze the conversion of arginine into citrulline in proteins. In the mammalian PAD family, approximately 50% of the amino acids are identical among different isoforms within one species, and 70% to 95% of the amino acids are identical among the same isoforms in different mammals [24]. Five isoforms of PAD (PAD I, PAD II, PAD III, PAD IV, and ePAD) have been cloned and sequenced in mice. PAD II is found in various tissues including skeletal muscle, uterus, spinal cord, salivary glands, and pancreas [43]. PAD I and PAD III are expressed in epidermis and hair follicles (PAD III) [43]. Mouse PAD IV has a potential nuclear localization sequence and is likely to be present in nuclei. ePAD has been localized in mammalian oocytes and embryos [26]. The isoform of PAD immunoprecipitated by ABL_2_, which is the isoform secreted from cortical granules, was only found in ovarian tissue.

PAD makes up about 1% of the total protein in mouse oocytes [26], and current data indicate that oocytes contain multiple isoforms of PAD. Immunohistological data obtained with three different PAD antibodies (anti-ePAD, anti-PAD-V, and ABl_2_) suggest mouse oocytes contain at least four isoforms of this protein. These isoforms are located in the nucleus (anti-PAD V), non-cortical cytoplasm (anti-PAD V), cortical cytoplasm (anti-ePAD) (current study and 27), and the cortical granules (anti-ePAD, anti-PAD-V, and ABl_2_). In an earlier study on p75 (now identified as PAD), whole oocyte extracts subjected to 2-dimensional gel electrophoresis revealed four species of p75 with pIs of 4.9 to 5.3 [20]. Likewise, a train of proteins designated PAD with pIs ranging from 5 to 5.5 was also observed in mouse oocytes [26]. These observations support the conclusion that mouse oocytes have at least four isoforms of PAD, which are localized in the cortical granules, cytoplasm (cortical and non-cortical), and nucleus of germinal vesicle intact oocytes.

The isoform found in the cortical granules is likely ePAD (or the egg and embryo abundant PAD, AAH53724) which is the only isoform predicted to be a secreted protein with a signal sequence by both the hidden Markov model (SignalP) and neural networks (TargetP) algorithms. ePAD was also predicted to be a non-transmembrane protein (data not shown) using TMHMM software, [44,45], which would be consistent with our observation that cortical granule PAD behaves as a peripheral membrane protein following exocytosis. While, several egg proteins without signal sequences have been identified on the extracellular surface of mouse oocytes [46], it is improbable that any of the PAD isoforms without signal sequences is the secreted isoform based on pI data. Of the four species of p75 (PAD) with pIs of 4.9 to 5.3 in mouse oocytes [20], only the one with a pI of 5.3 was released and detected in cortical granule exudates (Fig. 8D in [20]). Of the various PADs that could be present in oocytes, ePAD has a pI (5.36) which is most similar to the pI (5.3) of the secreted form of p75. From the above evidence, ePAD appears to be the best candidate for the cortical granule PAD.

It is probable that the nuclear PAD observed in germinal vesicle intact oocytes and preimplantation embryos is mouse PAD IV, which has a classic monopartite nuclear localization sequence motif (PPVKK_ST, Fig. 3 underline) in the same region as human PAD V [25]. Since human PAD V and mouse PAD IV are 70% identical in their amino acid sequence (Fig. 3), it is not surprising that polyclonal antibodies made against human PAD V react with mouse PAD IV immunocytochemically. Interestingly, the cytoplasmic immunoreactivity of the PAD antibody observed in the metaphase II oocytes was brighter than of that in the germinal vesicle intact oocytes, suggesting that the nuclear PAD IV became redistributed to the cytoplasm following germinal vesicle breakdown.

The cytoplasmic PAD that we observed in mouse oocytes is most likely PAD I, PAD II, PAD III, and/or the mouse PAD type V-like protein (XP_144067) since these isoforms are predicted to have neither a signal sequence nor nuclear localization sequence. Of these four isoforms, only the mouse PAD type V-like protein (XP_144067) has the VLIGGSFY sequence. ePAD was previously interpreted to be in sheets of intermediate filaments based on immunoelectron microscopic data using the ePAD antibody [26]. However, the ePAD antibody reacted in 2D gel electrophoresis with all isoforms of PAD and stained the nuclei, cortex, and interior cytoplasm of germinal vesicle intact oocytes [26], indicating that the antibody reacts with more than one isoform of PAD, as occurred with the anti-PAD V antibody in our study. Future studies will be necessary to fully identify and characterize the function of each of the PAD isoforms in oocytes.

Our data show that, following exocytosis, some cortical granule PAD remains associated with the plasma membranes as a peripheral protein. Interestingly, the ABL_2 _antibody recognizes a pair of mouse embryonic glycoproteins with approximate molecular weights of 65 and 70 kDa, that were localized to the cortical cytoplasm of preimplantation embryos at both the light and electron microscopic levels [15,38,39]. It is probable that the p65/p70 found in preimplantation embryos are different from the p75 (PAD) found in oocytes for several reasons. First p75 (PAD) is not glycosylated [20], while p65/70 are glycosylated [15]. Secondly, the molecular weights and the pIs of the two embryonic glycoproteins (65 to 70 kDa/pI = 6 to 7) and the oocyte protein p75 (75 kDa/pI = 4.9 to 5.3) are different [20,38]. Lastly, p65/70 are synthesized in a stage specific manner between the 2-cell and morula stages, while the synthesis of p75 (PAD) is detected in the early stages of oogenesis and increases during oocyte growth [38,39,47]. The above data support the conclusion that p75 and p65/70 are not identical. P75 (PAD) is present in the cortical granules of unfertilized oocytes, while p65/70 appear to be cytoplasmic proteins found in preimplantation embryos. It is possible p65/70 are cytoplasmic isoforms of PAD or that both the ABL_2 _and PAD antibodies cross react with another type of protein that shares an epitope (s) with the PAD family. In either case, the exact identity of p65/70 remains to be determined.

Most known substrates of PADs are either intermediate filaments or filament-associated proteins that have structural function. Recently, H3 and H4 histones were shown to be substrates of human PAD IV (equivalent to human PAD V) which is involved in regulating histone arginine methylation by converting methyl-arginine to citrulline [48]. In our study, cortical granule PAD appeared to play a role in early embryogenesis since PAD antibodies, but not control antibodies or a function blocking antibody to another oolemma protein (anti-β1 integrin), inhibited embryonic development. This finding agrees with two prior studies that documented the inhibitory effect of the ABL_2 _antibody on preimplantation development in mice and hamsters [16,20] and identifies a new potential function for PAD. Since PAD is a calcium dependent protein, it is possible that cortical granule PAD is activated by extracellular calcium when it is exocytosed at fertilization. The activated PAD could then citrullinate and consequently activate an extracellular mitogen(s) or membrane protein(s) that is involved in regulating early embryogenesis [49-52]. Alternatively, it is possible that the cortical granule PAD affects early embryogenesis by providing a microenvironment to protect the developing preimplantation embryos. For example, as a consequence of citrullination, the PAD target protein(s) may unfold, as occurs with PAD III, and thereby be rendered ready for cross-linking, which could form a more protective extracellular matrix in perivitelline space.

## Conclusion

Our study demonstrates that mouse oocytes contain multiple isoforms of PAD that are present in the nucleus, cortical cytoplasm, non-cortical cytoplasm, and cortical granules prior to fertilization. One isoform of PAD is released from the cortical granules at fertilization, and after its release, it remains associated with the zygote's and blastomeres' plasma membranes where it appears to play a role in preimplantation development.

## Authors' contributions

ML performed most the experiments and prepared the manuscript. AO and PT performed the experiments in plate 5. PC, MY, and SC provided the antibodies (ABL_2 _antibody, PAD V (N) antibody, and ePAD antibody, respectively) and critically reviewed the manuscript. PT supervised all the work and assisted in writing the manuscript.
